# Assessment of Vitamin D Status in the Drâa-Tafilalet Population (Morocco) Based on Sociodemographic, Health, and Nutritional Factors

**DOI:** 10.3390/nu16132118

**Published:** 2024-07-02

**Authors:** Fouzia Sebbari, Farid Khallouki, Ahmad Mohammad Salamatullah, Mohammed Bourhia, Amira Metouekel, Bachir El Bouhali

**Affiliations:** 1 Faculty of Science and Techniques, University Moulay Ismail of Meknes, Boutalamine, P.O. Box 509, Errachidia 52000, Morocco; 2 Department of Food Science & Nutrition, College of Food and Agricultural Sciences, King Saud University, P.O. Box 2460, Riyadh 11451, Saudi Arabia; 3 Laboratory of Biotechnology and Natural Resources Valorization, Faculty of Sciences, Ibn Zohr University, Agadir 80060, Morocco; 4 BOI R&D Laboratory, Bioval Ocean Indian Research and Innovation Company, 18 rue des Poivres Roses, 97419 La Possession, France; 5 Faculty of Science, University Moulay Ismail of Meknes, Zitoune, P.O. Box 11201, Meknes 50070, Morocco

**Keywords:** elderly, vitamin D, sociodemographic and health factors, Morocco

## Abstract

The purpose of this study was to evaluate the vitamin D status and determine the factors influencing it in the Drâa-Tafilalet community (southeastern Morocco). Sociodemographic factors, health, cognitive status, sun exposure, and nutritional conditions were examined to help us understand their association with vitamin D status. Vitamin D data were gathered through laboratory testing, while demographic and health information was collected through interviews with participants in 2023. The study involved 100 participants aged 60 and above, most of whom were women (85%) rather than men (15%). The majority of participants were Arabs (90%), with a minority being Amazigh (10%). The average vitamin D level was 31.83 ± 10.55 ng/mL, varying based on participants’ age, education, and gender. Sun-exposed individuals exhibited significantly higher mean vitamin D levels (33.56 ± 11.99 ng/mL) compared to those with limited sun exposure (28.97 ± 9.28 ng/mL). Moreover, the time spent outdoors, seasonal changes, and the duration of sun exposure affected the levels of vitamin D. These findings depict the vitamin D status of the elderly population of Drâa-Tafilalet, recognized as one of Morocco’s poorest regions, shedding light on the significant influencers. Nonetheless, additional research is necessary to explore the correlation between dietary habits, sunlight exposure, and vitamin D levels in both young and elderly populations.

## 1. Introduction

Vitamin D, a fat-soluble vitamin, serves as both a hormone produced by the human body when exposed to UV-B rays from the sun and a dietary component [1,2]. Vitamin D exists in two forms depending on its origin: type D2, named ergocalciferol, produced by plants, and type D3, or cholecalciferol, produced by animals and certain lichens [3,4]. These two molecules are 9, 10-secosteroids. The human body also synthesizes vitamin D3 in the skin under the effect of the ultraviolet rays of the sun [5,6]. The vitamin D receptor is a nuclear receptor and a fat-soluble secosteroid [7]. The regulation of over 3% of the human genome is carried out by this hormone system [8,9]. More recently, however, low vitamin D levels have been linked to several debilitating diseases, including diabetes, autoimmune disorders, depression, cancer, and cardiovascular and renal diseases, and an increased risk of falls [10,11,12]. Initially, vitamin D was recognized for its role in phosphate and calcium metabolism [13]. According to Rai and Agrawal (2017) [14], this vitamin sustains mineral balance and skeletal health. Given its steroid-like nature, vitamin D is essential for heart health, especially when it comes to deficiency [15].

Approximately 80% of the vitamin D absorbed by humans results from cutaneous synthesis induced by sunlight, while the remaining 20% is acquired through diet and supplements [10,16]. For example, ergocalciferol (D2) is derived from plant sources, while cholecalciferol (D3) comes from animal sources [17]. Notwithstanding the differences in their sources, these two compounds go through comparable metabolic processes [18]. Furthermore, a vitamin D binding protein (DBP) transfers these precursors to the liver, where a specific hydroxylase produced by the *CYP2R1* gene hydroxylates vitamin D at the C25 position [19,20]. Moreover, the primary form of vitamin D that circulates is 25-hydroxyvitamin D (25(OH)-D) [21]. Although it has been thought of as a precursor to the active form, 1,-25-(OH)-2D, it can bind to the vitamin D receptor (VDR) at very high doses. Moreover, megalin is expressed by a variety of extrarenal cells, including osteoblasts, parathyroid cells, and 1α-hydroxylase [22]. Consequently, various tissues produce 1,-25-(OH)-2D locally [10].

Vitamin D is typically regarded as the elusive vitamin in human diets due to its scarcity in food [23]. Similarly, although wintertime UV-B radiation is sufficient for the synthesis of vitamin D in many parts of the world, lifestyle choices and worries about the possible health effects of sun exposure can result in inadequate exposure and a deficiency in vitamin D [24]. Under these conditions, maintaining optimal vitamin D status requires consuming foods or supplements high in the vitamin [25]. However, there is a significant danger of vitamin D insufficiency without regular exposure to sunlight or fortified foods. Thus, factors including age, lifestyle, geography, environment, and skin pigmentation have an impact on vitamin D synthesis [26]. The Workshop Consensus for Vitamin D Nutritional Guidelines states that over 50% of the elderly population has vitamin D levels that are insufficient or inadequate [10]. Even in regions with abundant sunshine, this holds true for younger populations. As a result, it is estimated that over a billion individuals worldwide have insufficient levels of vitamin D [27].

Numerous studies have examined various topics related to vitamin D in various zones of Morocco [28,29,30,31]. Field surveys have addressed the status of vitamin D among healthy-aged populations [28], Moroccan pregnant women and newborns, and school-aged children in rural regions [30]. In the laboratory, researchers have addressed vitamin D (D3) status among the population of eastern Morocco with high-performance liquid chromatography [31], the relationship between bone density and vitamin D status in systemic scleroderma [32], and the connection between ankylosing spondylitis disease activity, vitamin D levels, and bone mineral density [33]. The results of these investigations recorded the deficiency of vitamin D in different categories of Moroccan populations in both urban and rural areas. However, more investigations are needed to clarify the effect of public health status, nutritional state, and other factors on the level of vitamin D.

Based on the fragmentary data on vitamin D in Morocco, this study aimed to examine the level of hydroxyvitamin D among the elderly population of Drâa-Tafilalet, located in southeast Morocco. Further, this research investigated the variability of hydroxyvitamin D depending on various factors, including sociodemographic features, health status, and the nutritional state of the participants. This study aimed to explore the status of vitamin D among the eastern population of Morocco, known for its Saharan climate and high exposition to sunlight. This is the first investigation into vitamin D specifically focusing on its implications for public health, nutrition, and sociodemographic aspects.

## 2. Materials and Methods

### 2.1. Study Area

This study was conducted in the region of Drâa-Tafilalet, located in the southeast zone of Morocco. The Drâa-Tafilalet region (31.41° N latitude) covers an area of 88,836 km^2^ and includes five provinces: Errachidia, Midelt, Ouarzazate, Tinghir, and Zagora. This region is bordered by four regions: Oriental in the northeast, Fez-Meknes in the north, Beni Mellal-Khenifra in the northwest, Souss-Massa in the southwest, and the Algerian–Moroccan border to the east. The population of the region is estimated to be 1,635,008 people.

The climate in the Drâa-Tafilalet region displays diversity owing to its broad spectrum of ecosystems. This region geographically stands for two main zones: the High Atlas Mountains in the northern area and green oases encircled by the Anti-Atlas Mountains in the south. The High Atlas Mountains soar to over 3000 m, experiencing snowfall in the winter. In contrast, the green oases and Anti-Atlas Mountains endure an arid climate, with summer temperatures reaching up to 48 °C.

### 2.2. Subjects and Study Design

For this cross-sectional study, 100 elderly volunteers (over 60 years) were consulted at urban and rural healthcare infrastructures. The study excluded participants with thyroid, liver, or kidney issues. Two elderly people refused to give a blood sample, and eight women refused to take part in this study. Therefore, they were removed from the pool of participants.

### 2.3. Pilot Sample

Before the survey, the questionnaire was administered to a pilot sample of 45 people from the same population, who were not included in the study. Minor modifications were made to the questionnaire to clarify misunderstood questions.

### 2.4. Data Collection

The data of this study were gathered during the summer of 2023. Face-to-face interviews were conducted using an organized methodology. Gender, age, ancestry, education level, monthly income, employment, health characteristics, daily sun exposure, bedsores, nutritional status as determined by the Mini Nutritional Assessment (MNA) [34,35], body mass index (BMI) [36], and the elderly’s cognitive and depression states were among the information gathered. Gender was divided into men and women, while education level ranged from illiterate to university level. Ancestral origin was divided into two categories, Arabs and Amazigh, based on the ethnic characteristics of Morocco and the whole of North Africa. Marital status was divided into four categories: Single, Married, Widow, and Divorced. The professions of the interviewed populations were divided into With, Without, and Retired. Monthly income was divided into four levels, ≤2000, 2000–4000, 4000–8000, and >8000, based on the economic situation of local populations in the study area. In terms of daily sun exposure, the questions addressed the exposure (exposed or not), period of exposure (hours), exposure manner (direct or indirect), and exposure season (spring, summer, autumn, or winter). Body mass index was divided into four categories: Underweight (BMI < 21), Normal Weight (BMI 21–24.99), Overweight (BMI ≥ 25), and Obesity (BMI = 30–40). These parameters were taken into consideration during the measurement of hydroxyvitamin D to help clarify their effect on the level of hydroxyvitamin D.

Based on weight and height data, the body mass index (BMI, kg/m^2^) was computed (weight was assessed with little clothing with an accuracy of ±50 g). The condition of protein-energy malnutrition in the elderly was determined by a BMI below 21 kg/m^2^. The nutritional status was evaluated using the Mini Nutritional Assessment (MNA). Cognitive assessment was conducted through the Arabic version of the Mini Mental Status Examination (MMSE), which was adapted and translated by Dr. M. El Alaoui Faris, L. Ettahiri, and F. Ben Belaid from the Neurological Department and Neuropsychological, Specialized Hospital of Rabat. The Mini International Neuropsychiatric Interview (MINI), in its Moroccan Arabic Version 5.0.0, identified the subjects’ depressive states. This structured diagnostic interview (MINI- DSM-IV) is standardized and explores a wide range of DSM-IV Axis I psychiatric disorders.

### 2.5. Laboratory Assessments

The vitamin D assay performed in this study used a competitive immunodetection method [37]. In this method, the target material in the sample (25(OH)-D) is bound to a labeled antibody (fluorescence) in the detection buffer to form a complex mixture. The complex is then directed to migrate into a nitrocellulose matrix where the covalent synthesis of 25(OH)-D is immobilized on the test strip and interferes with the binding of the target material and the labeled antibody. Consequently, the more target material present in the blood, the fewer detection antibodies accumulate, resulting in a lower fluorescence signal.

On the same day as collection (sampling day), serum 25(OH)-D levels were measured. The amount of hydroxyvitamin D [25(OH) D2/D3] was measured using an Ichroma™ reader (Boditech Med Inc., Chuncheon, Republic of Korea) with a measurement range of 8–70 ng/mL.

The Ichroma™ Vitamin D check can be considered an objective assessment of the accuracy of Ichroma™ Vitamin D as part of the machine’s quality testing and calibration. This control was supplied in freeze-dried form with 150 µL of sterilized distilled water. The following categories applied to the 25(OH) D levels. It was determined that 25(OH) D levels were severely deficient if they were less than 10 ng/mL, insufficient if they were between 10 and 29.99 ng/mL, and normal if they were more than 30 ng/mL.

The sampling and measurements were carried out following the international and national (Morocco) standards. The sampled blood was taken following the standards and conserved under adequate conditions.

### 2.6. Statistics

After field prospection and laboratory measurements, Excel was used to arrange the gathered data for every parameter. Moreover, percentages of sociodemographic, health, and nutritional parameters were calculated. The mean levels of hydroxyvitamin D and the age of participants (N = 100) were noticed. The comparison of hydroxyvitamin D levels between men and women (gender), Arab and Amazigh (origin), depressive and non-depressive, hospitalized and non-hospitalized, suffering and healthy, and medicated and non-medicated individuals was performed using a simple *t*-test. Multiple range tests were conducted to compare the hydroxyvitamin D levels among the different age categories, sunlight exposure, and education levels of the participants.

## 3. Results

### 3.1. Features of Participants

The sociodemographic characteristics of the interviewed participants are presented in Table 1. A total of 100 participants were investigated in the study. Women participants dominated, accounting for 85%, while men accounted for only 15%. Among the participants, 49% were widows and 48% were married, while divorced and single individuals represented only 2% and 1%, respectively. The majority of the surveyed group, around 40%, consisted of illiterates, autodidacts represented 35%, and those with a primary-school-level education comprised 19%. In contrast, participants with secondary-school- and university-level education made up only 3% each. In terms of professions, three types were recorded among participants; the majority were unemployed (82%), 15% were employed, and retirees constituted 3% of the group. The ethnic origin of the interviewed people was divided into two types, Arabs, who were predominant, comprising 90%, and Amazigh, represented by 10%. The age of the participants was 60 years and older, with a mean of 69.65 ± 7.83 years.

The interviewed participants were divided into different categories of monthly incomes. The lowest income (≤ MAD 2000) was recorded in 26% of participants, and 61% had monthly incomes of between MAD 2000 and 5000, while 10% reported earning between MAD 5000 and 8000 per month. A monthly income of more than MAD 8000 was recorded among 3% of participants.

### 3.2. Status of Vitamin D

**Figure 1:**
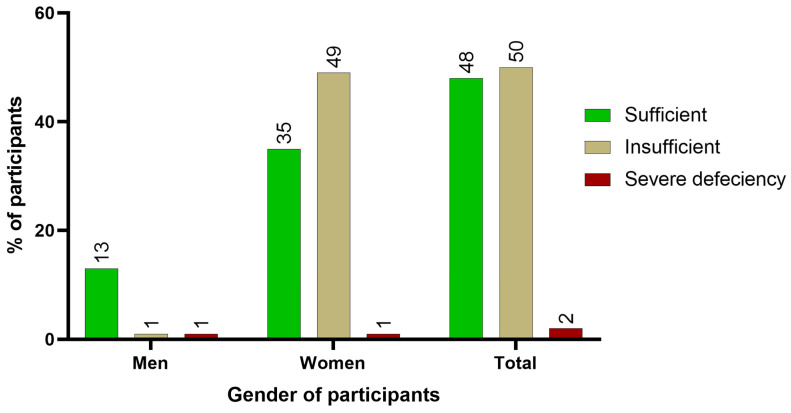
Status of vitamin D among the elderly of Drâa-Tafilalet by gender.

The status of vitamin D among the participants interviewed in Drâa-Tafilalet is presented in Figure 1. The majority of participants (50%) had insufficient levels of hydroxyvitamin D, with 49% of them being women. In contrast, 48% of the sampled population had sufficient levels. A smaller portion of participants had severely deficient levels of hydroxyvitamin D.

**Figure 2:**
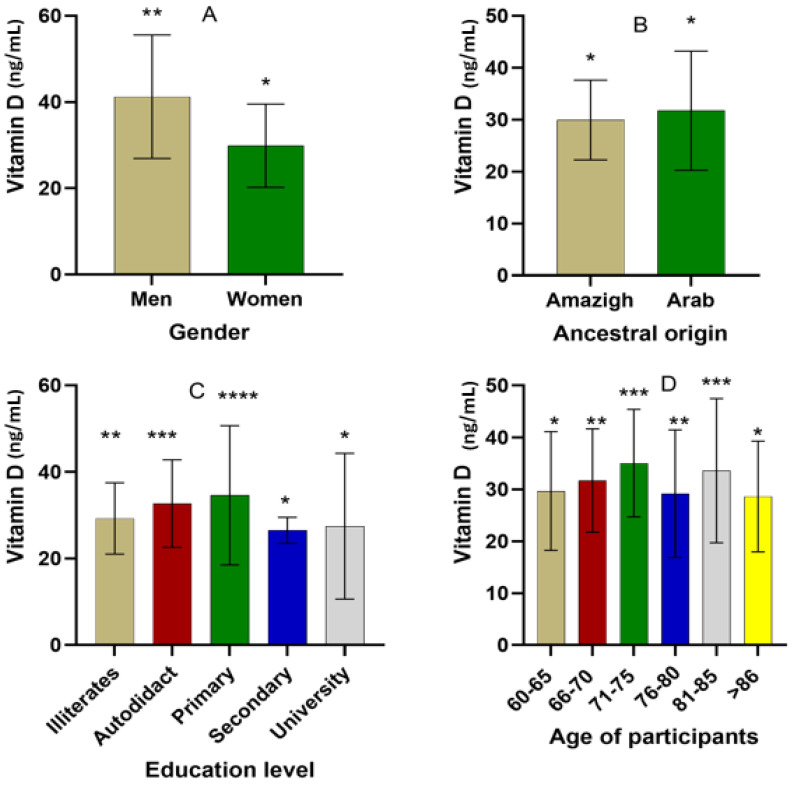
Variation in hydroxyvitamin D levels depending on the demographic features of participants ((**A**) gender; (**B**) ancestral origin, (**C**) education level; and (**D**) age categories) (* denotes statistical differences between compared groups at different *p*-values; * *p* < 0.05; ** *p* < 0.01; *** *p* < 0.005; **** *p* < 0.001).

The variation in hydroxyvitamin levels based on demographic features is shown in Figure 2. Regarding gender, there was a statistically significant difference in hydroxyvitamin D levels between women (29.87 ± 9.63 ng/mL) and men (41.24 ± 14.31 ng/mL) among the interviewed participants (*p* < 0.05). Hydroxyvitamin D levels were significantly higher among participants of Arab descent (31.75 ± 11.49 ng/mL) compared to Amazigh participants (29.96 ± 7.69 ng/mL). However, hydroxyvitamin D levels varied depending on the education level and age of the participants. The highest hydroxyvitamin D values were recorded among primary- (34.60 ± 16.10 ng/mL) and self-educated participants (32.72 ± 10.09 ng/mL), followed by among illiterates (29.28 ± 8.27 ng/mL). In contrast, hydroxyvitamin D levels were statistically similar between participants with a university degree (27.46 ± 16.86 ng/mL) and those with a secondary education (26.52 ± 3.01 ng/mL). In terms of age, the highest hydroxyvitamin D values were observed among the elderly aged 71–75 and 81–85 years old (35.06 ± 10.32 and 33.59 ± 13.88 ng/mL, respectively), followed by among participants aged 60–65 and 66–70 years old (29.70 ± 11.43 and 31.71 ± 9.96 ng/mL, respectively). On the other hand, the lowest levels of hydroxyvitamin D, estimated to be 29.19 ± 12.29 and 28.64 ± 10.68 ng/mL, were recorded among participants aged 76–80 and over 86 years old.

### 3.3. Health Status of Participants

The health status of the participants interviewed is detailed in Table 2. It is worth noting that 95% of the participants were found to be in a non-depressed state, while only 5% experienced depression. In terms of illnesses, 48% of the participants had various health conditions, while 52% were not affected. Out of this population, 54% of the participants reported having been hospitalized at some point in their lives. Additionally, 53% of the participants underwent medication for their illnesses, while 47% did not. Insomnia was reported by 25% of the participants, while the remaining 75% were not affected by this condition.

**Figure 3:**
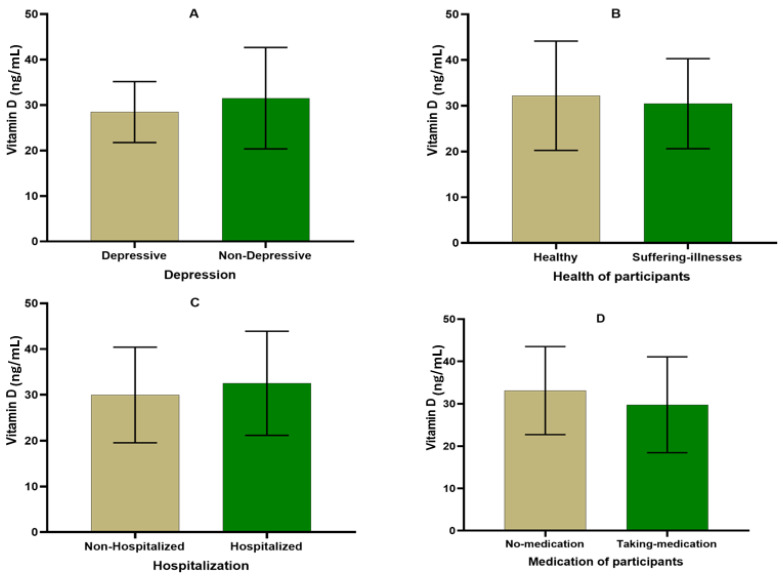
Variation in hydroxyvitamin D levels depending on the health status of participants ((**A**) depression; (**B**) diseases; (**C**) hospitalization; and (**D**) medication).

The variation in hydroxyvitamin D levels depending on participants’ health status is presented in Figure 3. Among participants affected by depression, the hydroxyvitamin D level was estimated to be 28.47 ± 6.70 ng/mL compared to 31.51 ± 11.15 ng/mL in non-affected participants (*p* > 0.05). The value of hydroxyvitamin D among healthy participants was estimated to be 32.19 ± 11.94 ng/mL, and, in participants who suffered from illnesses, it was 30.45 ± 9.85 ng/mL (*p* > 0.05). Furthermore, non-hospitalized participants recorded an estimated value of hydroxyvitamin D of 29.98 ± 10.44 ng/mL, which was statistically similar to the 32.53 ± 11.36 ng/mL recorded among hospitalized participants. Vitamin D values were similar in participants who took medication (29.77 ± 11.32 ng/mL) compared to those who did not take medication (33.14 ± 10.38 ng/mL).

### 3.4. Cognitive Status

**Figure 4:**
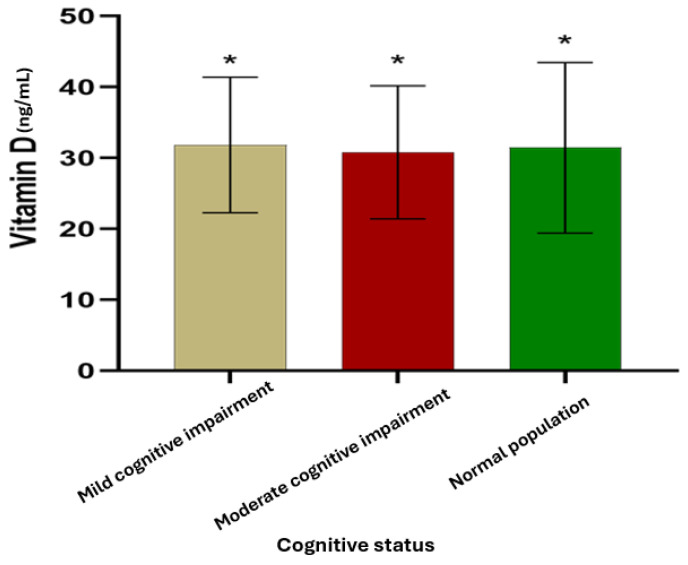
Level of hydroxyvitamin D among interviewed participants from Drâa-Tafilalet (* denotes statistical similarity at *p* < 0.05).

The basic characteristics of the participants’ cognitive status are presented in Table 3. There were four categories used to classify the participants’ cognitive status: normal, mild cognitive impairment, moderate cognitive impairment, and severe cognitive impairment. Each category represented a different percentage of the individuals who were interviewed. Most interviewed participants (59%) were not affected, 26% had mild cognitive impairment, and 15% had moderate cognitive impairment. Severe cognitive impairments were not recorded among participants. The estimated hydroxyvitamin D level in individuals with mild cognitive impairment was 31.84 ± 9.56 ng/mL. In participants with moderate cognitive impairment, the hydroxyvitamin D level was estimated to be 30.81 ± 9.38 ng/mL. Furthermore, the hydroxyvitamin D level among non-affected populations was recorded as 31.45 ± 12.02 ng/mL (Figure 4).

### 3.5. Nutritional Status and Body Weight Index

Table 4 illustrates the results relating to the participants’ nutritional status and body mass index. Among these individuals, three nutritional statuses were documented, each with varying percentages. Satisfactory nutritional status was observed in 72% of the participants, while 28% were at risk of malnutrition. Severe malnutrition was not documented in any of the participants. As for the body mass index, only 20% of the participants had a normal weight, while 9% were underweight. In contrast, 35% of the participants exhibited overweight status, and another 36% were affected by obesity.

**Figure 5:**
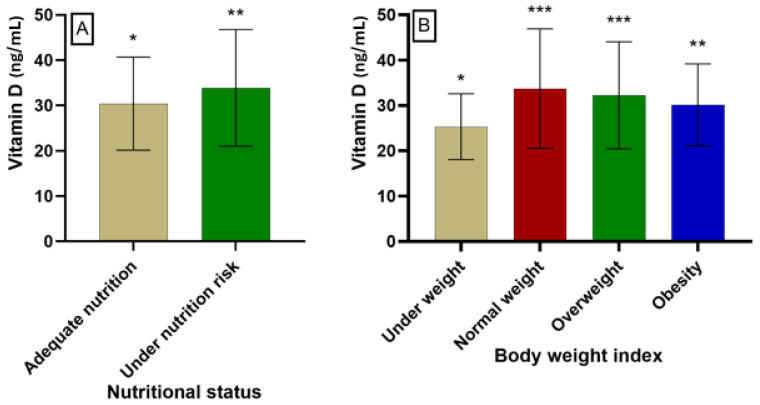
Level of hydroxyvitamin D depending on nutritional (**A**) and body weight (**B**) status of participants (* denotes statistical difference between compared groups at different *p*-values; * *p* < 0.05; ** *p* < 0.01; *** *p* < 0.005).

The hydroxyvitamin D level was estimated to be 30.41 ± 10.27 ng/mL among participants with satisfactory nutrition (Figure 5). Participants at risk of malnutrition had a hydroxyvitamin D level of 33.88 ± 12.87 ng/mL, while those who were underweight had a level of 25.33 ± 7.26 ng/mL. Conversely, participants with normal body weight had a hydroxyvitamin D level equal to 33.74 ± 13.18 ng/mL. In participants who were overweight and obese, the levels of hydroxyvitamin D were 32.26 ± 11.81 and 30.16 ± 9.01 ng/mL, respectively.

### 3.6. Effect of Sun Exposition on Vitamin D

**Figure 6:**
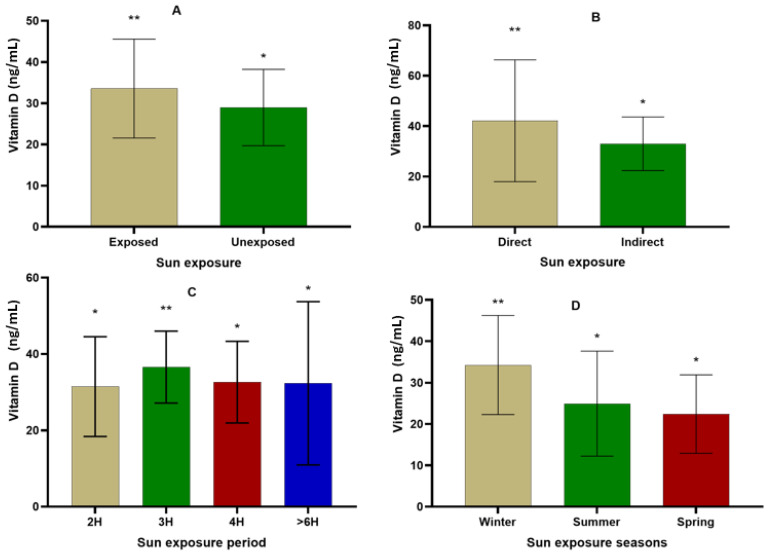
Variation in hydroxyvitamin D level depending on sun exposition (**A**), type of exposition (**B**), period of exposition (**C**), and seasons of exposition (**D**) (* *p* < 0.05; ** *p* < 0.01).

Figure 6 presents the variation in hydroxyvitamin D levels depending on sun exposure (A), type of exposure (B), period of exposure (C), and seasons of exposure (D). The hydroxyvitamin D level was 33.56 ± 11.99 ng/mL in participants exposed to the sun compared to 28.97 ± 9.28 ng/mL in participants not exposed (*p* < 0.001). Participants exposed directly to sunlight showed a level of hydroxyvitamin D estimated to be 42.13 ± 24.19 ng/mL compared to 33.00 ± 10.64 ng/mL in participants exposed to sunlight through an indirect method (*p* < 0.001). In terms of exposure period, the values of hydroxyvitamin D were statistically similar between exposure periods of 2 h (31.47 ± 13.05 ng/mL), 4 h (32.64 ± 10.67 ng/mL), and > 6 h (32.33 ± 21.40 ng/mL). However, the highest value of hydroxyvitamin D (36.58 ± 9.40 ng/mL) was recorded in participants with 3 h of sun exposure. Furthermore, the level of hydroxyvitamin D was significantly higher in participants exposed to sunlight during winter (34.28 ± 11.96 ng/mL) compared to summer (24.93 ± 12.68 ng/mL) and spring (22.41 ± 9.49 ng/mL).

## 4. Discussion

Insufficient vitamin D levels are a problem for approximately 50% of people worldwide [38,39]. Across all ages and ethnicities, an estimated 1 billion people globally suffer from vitamin D insufficiency (VDD) [40]. The main causes of this hypovitaminosis D pandemic are various lifestyle and environmental factors that limit sunlight exposure, which is necessary for UVB-induced vitamin D production in the skin [41,42]. These factors include reduced outdoor activity and air pollution. In this study, we investigated the vitamin D status and the factors affecting it among the population of Drâa-Tafilalet. The obtained data provided new insights into the vitamin D status among the elderly. Additionally, we demonstrated the impact of demographic features, health status, cognitive status, and nutritional aspects on hydroxyvitamin D levels. These findings are the first of their kind in Morocco and are highly relevant to research efforts in public health, particularly regarding insufficient vitamin D.

Furthermore, in this study, we characterized the sociodemographic characteristics of the population in Drâa-Tafilalet, Morocco. The major participants were women, with widows and married individuals being the most common. Similarly, among the interviewed participants, analphabets and autodidacts were the most frequently recorded. Several field and laboratory studies have addressed vitamin D among the Moroccan population, yielding variable results [29,31,32,43]. Skalli et al. (2018) [29] examined the association between vitamin D status and multiple sclerosis in Morocco’s population and found that, among 113 patients with the disease and 146 healthy individuals, 75% were women. The ages of the cases ranged from 18 to 60 years, while the ages of the controls were 33.6 ± 11.3 years and 36.8 ± 10.8 years, respectively. In another study, Allali et al. (2009) [44] investigated the prevalence of hypovitaminosis D in Morocco and its relationship with lifestyle, physical performance, bone markers, and bone mineral density. All participants in this study were women, with an average age of around 50 ± 9.3 years and a range from 40 to over 55 years old. Der Meer et al. (2011) [43] examined the prevalence of vitamin D deficiency among Turkish, Moroccan, Indian, and sub-Saharan African people in Europe and their countries of origin to compare it with that of other communities around the Mediterranean basin. The participants were from Turkey, Morocco (local and European immigrants), and India, and they were divided into men and women, with varying percentages depending on the targeted countries. The population from Turkey was divided into women (n = 126) and men (n = 87), while the Moroccan samples were composed only of men, and men were not recorded among the participants [43]. The age range of participants was 27 to 37 years old for women and 18 to 69 years old for men from Turkey. In Morocco, the age of the participants was 38 to 52 for men and 43 to 56 for women. In our case, the mean age of participants was 69.65 ± 7.83, which agrees with the cited results. Recently, Lhilali et al. (2023) [45] investigated vitamin D levels in Moroccan women of childbearing age and their relationship with the sun exposure score. In total, 160 women between the ages of 18 and 45 were interviewed, with a median age of 25 years. Furthermore, 50% of the participants were housewives, 35% were students, and 15% were officials/salaried. In our instance, 82% of interviewees had no profession, 15% were employed, and 3% were retired. This study evaluated the status of vitamin D among the population of Drâa-Tafilalet. Bour and Nejjar (2017) [46] investigated the prevalence of hypovitaminosis D among the Moroccan population and recorded different values depending on the participants’ gender. The obtained results showed that the rate of this deficiency was estimated to be 85.2% in men and between 78.1% and 98.4% in women. The prevalence fluctuated between 70.1% and 90% among patients consulting in ambulatory medicine. In another study, Lhilali et al. (2023) [45] investigated the level of vitamin D in populations of urban and rural areas of Meknes in central Morocco. The interviews were conducted with 160 women of childbearing age, ranging from 18 to 45 years old, and the results revealed that the women were affected by insufficient vitamin D, which is influenced by various factors, including sun exposure. Additionally, it is important to note that diet is the main source of external vitamin D. Therefore, improper nutrition is believed to influence vitamin D levels. In this case, further investigation is needed to understand the dietary aspect relevant to the population of Drâa-Tafilalet.

Dadda et al. (2021) [47] evaluated the vitamin D status among populations of the Drâa-Tafilalet region in eastern Morocco. Data were collected from 331 adults visiting local healthcare units during the summers of 2019 and 2020, and the results revealed that the prevalence of vitamin D deficiency was 37.5%, while that of vitamin D insufficiency was 56.5%. Bouaddi et al. (2014) [48] recorded hypovitaminosis D in 75% of patients (children with juvenile idiopathic arthritis) in the Rabat-Sale region. In a study conducted on 331 adults in the Drâa-Tafilalet region, the value fluctuated between 8 and 17.9 mg/mL, with a mean of 11.8 mg/mL [47].

In another study conducted by Skalli et al. (2018) [29], the levels of hydroxyvitamin D were compared between healthy participants and patients affected by multiple sclerosis in Rabat (west of Morocco) from January 2012 to March 2015. The results showed that the value of vitamin D was estimated at 11.69 ± 6.97 ng/mL in patients and 12.98 ± 6.58 ng/mL in controls.

Numerous studies have explored the relationship between vitamin D levels and sunlight exposure, yielding varying results [5,45,49]. In Morocco, this is the first study to investigate the impact of sunlight exposure on hydroxyvitamin D levels among the populations of the eastern oases in Drâa-Tafilalet. In other Moroccan studies, Lhilali et al. (2023) [45] examined the relationship between sun exposure score and vitamin D levels in women of childbearing age in Meknes city, central Morocco. The study included 160 women aged 18 to 45 years and found a significant effect of sun exposure on vitamin D levels. The study also revealed a significant association between serum vitamin D levels and the total sun exposure score. However, it was observed that the type of exposure (indoors or outdoors) had an impact on vitamin D levels. In our case, individuals exposed to sunlight had notably higher vitamin D levels. Similar results were reported by Aydın et al. (2019) [50] in a study conducted on 555 elite-level sportsmen aged 5 to 52 years old, as well as by Krzywanski et al. (2016) [51] for Polish elite athletes. These authors confirmed that participants with outdoor sunlight exposure had increased vitamin D levels. Similarly, Krzywanski et al. (2016) [51] found a positive association between vitamin D levels and sunlight exposure, which is in agreement with our findings. These authors recorded higher levels of vitamin D during the summer, while, in our results, the highest level of vitamin D was found during the winter. In our case, the populations were exposed to more sunlight during winter because the summer in Drâa-Tafilalet is very hot and dry. Therefore, populations stay at home.

## 5. Conclusions

Vitamin D is considered notably as the missing vitamin in humans due to its scarcity in their diet. While UV-B exposure is crucial, dietary intake from foods or supplements plays a pivotal role in maintaining optimal levels of hydroxyvitamin D. As is well known, the lack of regular sunlight exposure or fortified foods creates a potential risk of vitamin D insufficiency. This study assessed the vitamin D status within the Drâa-Tafilalet population in southeast Morocco. The findings underscore the significance of vitamin D inadequacy, particularly among the elderly. Various demographic factors such as age, gender, and income significantly influenced participants’ levels of hydroxyvitamin D. Moreover, the participants’ health status, including ailments, hospitalizations, and medication usage, influenced their levels of hydroxyvitamin D. The nutritional status of the participants also played a part, with those with satisfactory nutritional status being less susceptible to vitamin D deficiency.

These results are of paramount importance for subsequent scientific investigations, serving as a reference for research on public health and nutrition in North Africa. Furthermore, these data could offer invaluable information to Moroccan policymakers, particularly for addressing health challenges.

However, we understand that, due to the limited number of participants and the study period being in the spring, the predictive value of the current findings needs to be validated in other experimental conditions with a larger population (including women and men) at different latitudes/altitudes and during different seasons of the year.

## Figures and Tables

**Table 1:** Sociodemographic features of participants from Drâa-Tafilalet.

Percentage or Mean	Parameters	
69.65 ± 7.832	Age (years)	
85 (85%)	Women	Gender
15 (15%)	Men	
90 (90%)	Arab	Ancestor origin
10 (10%)	Amazigh	
1 (1%)	Single	Marital situation
48 (48%)	Married	
49 (49%)	Widow	
2 (2%)	Divorced	
40 (40%)	Illiterate	Scholar level
35 (35%)	Autodidact	
19 (19%)	Primary	
3 (3%)	Secondary	
3 (3%)	University	
15 (15%)	With	Profession
82 (82%)	Without	
3 (3%)	Retired	
26 (26%)	≤2000	Monthly income (MAD)
61 (61%)	2000–5000	
10 (10%)	5000–8000	
3 (3%)	>8000	

**Table 2:** Variation in health status among interviewed participants from Drâa-Tafilalet.

Percentages	Parameters	
5 (5%)	Depressive	Depression
95 (95%)	Non-depressive	
48 (48%)	Yes	Sufferance of diseases
52 (52%)	No	
54 (54%)	Yes	Hospitalization
46 (46%)	No	
53 (53%)	Yes	Medication
47 (47%)	No	

**Table 3:** Variation in cognitive status among interviewed participants from Drâa-Tafilalet.

Percentages	Parameters	
59 (59%)	Normal	Cognitive status
26 (26%)	Mild cognitive impairment	
15 (15%)	Moderate cognitive impairment	
0 (0%)	Severe cognitive impairment	

**Table 4:** Nutritional status and body weight index among interviewed participants from Drâa-Tafilalet.

Percentages	Parameters	
72 (72%)	Adequate nutritional status	Nutritional status
28 (28%)	Risk of malnutrition	
0 (0%)	Severe malnutrition	
9 (9%)	Underweight (<21)	Body mass index (kg/m^2^)
20 (20%)	Normal weight (21 to 24.99)	
35 (35%)	Overweight (≥25)	
36 (36%)	Obesity (30–40)	

## Data Availability

Data are available upon reasonable request.
